# Life cycle assessment of the production processes for high-value biomass derivatives HMF and FDCA

**DOI:** 10.1038/s41598-026-39916-3

**Published:** 2026-02-12

**Authors:** Yang Gao, Qingyu Liu, Huanhuan Wei, Yungong Hu

**Affiliations:** 1 College of Engineering, Shenyang Agricultural University, Shenyang, 110866 China; 2 Henan Shuanghui Investment & Development Co., Ltd., Luohe, 462000 China

**Keywords:** 5-Hydroxymethylfurfural, 2,5-furandicarboxylic acid, Life cycle assessment, High value biomass utilization, Straw, Energy science and technology, Environmental sciences

## Abstract

The production of high-value-added products from biomass is a key strategy for advancing carbon neutrality. This approach not only reduces dependence on fossil resources but also enhances the economic value and utilization efficiency of renewable materials. 5-hydroxymethylfurfural (HMF) and 2,5-furan dicarboxylic acid (FDCA) are potential candidates for producing high-value-added chemicals via carbon-neutral pathways. This study conducts life cycle assessment (LCA) in accordance with ISO 14,040/44 standards, defining the functional unit as 1 ton of straw (for HMF production) and 284.34 kg of HMF (for FDCA production). Results show HMF_straw_ outperforms HMF_fructose_ in all categories, reducing 87.73 kg CO₂ eq and 7.87 kg 1,4-DB eq per unit product. HMF_fructose_ has 23.46%-27.83% higher aquatic/sediment ecotoxicity. Sensitivity analysis indicates that replacing the existing power structure (60% coal-fired power + 40% renewable energy) with 100% renewable energy can reduce global warming potential GWP by 74.56%. Replacing dichloromethane (DCM) with γ-valerolactone (GVL) reduces HT by 63.36%. Crystallization for FDCA is more sustainable than distillation, reducing abiotic depletion, acidification, human toxicity and photochemical oxidation by 50.22%-59.02%, and ~ 20% in fossil energy and global warming potential. The results confirm that straw is an environmentally viable feedstock for HMF production, crystallization represents more sustainable pathway for FDCA synthesis, and optimizing the power structure alongside solvent substitution can significantly reduce environmental impacts. This provides quantifiable reference criteria for green optimization in biomass chemical processes.

## Introduction

**Fig. 1:**
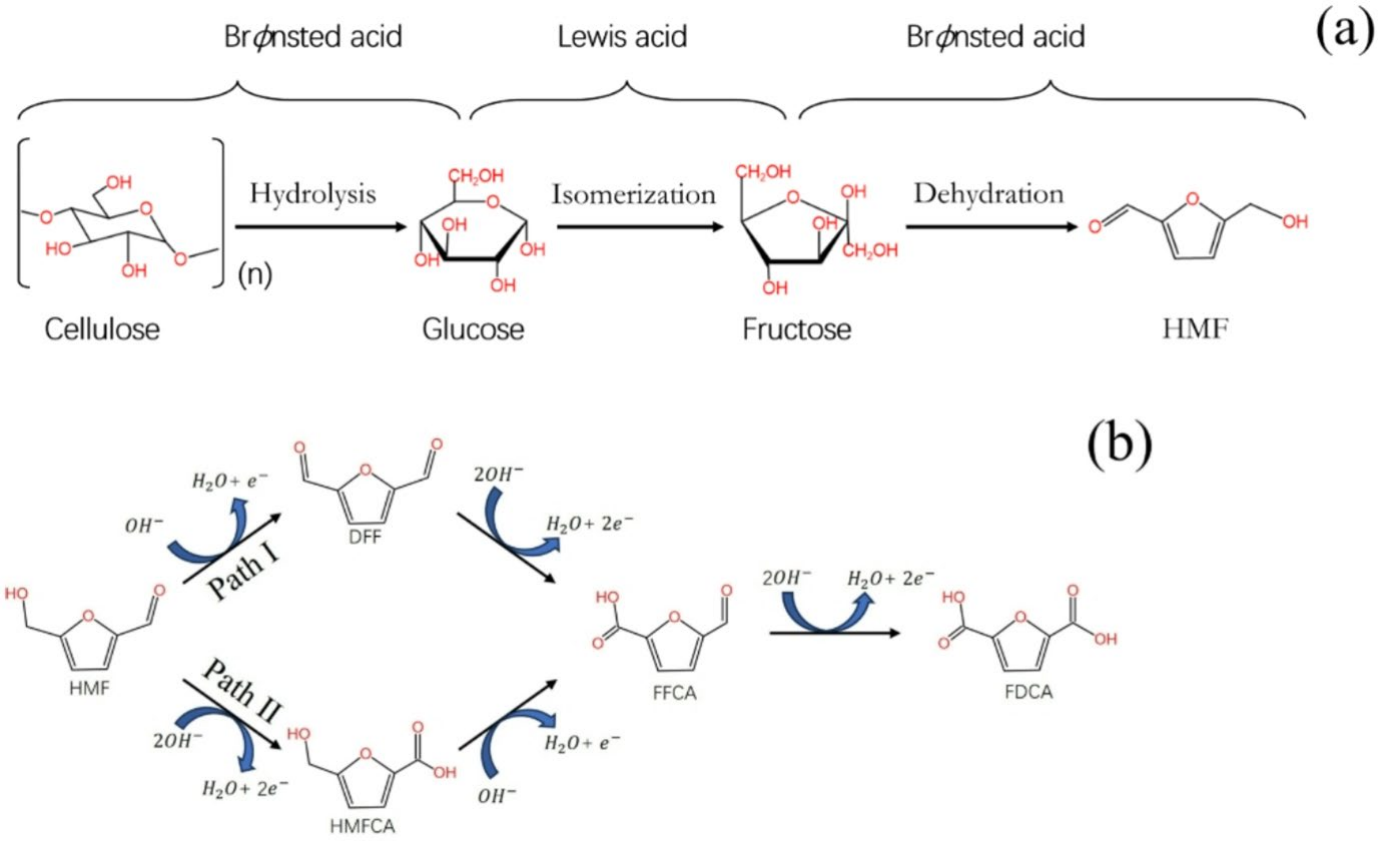
Reaction mechanism of HMF preparation from cellulose **(a)**; reaction principle of FDCA from HMF oxidation **(b)**.

Biomass, the fourth largest energy source after oil, coal, and natural gas, is the only renewable carbon-containing resource, offering numerous advantages such as abundant availability, wide geographic distribution, zero carbon dioxide emissions, and waste recyclability^1–5^. Globally, biomass is plentiful; in China alone, the annual output of crop straw reaches 865 million tons, providing substantial support for global sustainable development^6–10^. Unlike fossil fuels, biomass absorbs carbon dioxide during growth and achieves near-zero emissions when converted into chemicals, thereby helping to mitigate the impact of climate change^3,4,11–13^. A diverse range of biomass sources, including lignocellulose, oils and fats, and starch, can be transformed into high value-added chemicals through various conversion technologies such as pyrolysis, electrocatalytic conversion, and chemical catalytic conversion^14–16^. Among the main products of biomass dehydration are furans, with 5-hydroxymethylfurfural (HMF) being the most prominent due to its critical role as a precursor in the production of plastics, biofuels, and pharmaceuticals (Fig. 1(a))^1,17–20^. HMF is gaining considerable attention in recent biomass research due to its sustainability, ease of handling, and low cost. Its direct oxidation yields 2,5-furandicarboxylic acid (FDCA), recognized as one of the twelve platform chemicals for the green chemical industry (Fig. 1(b))^1,21–25^. As a core compound in the furan family, FDCA holds significant potential to replace fossil-based terephthalic acid in the synthesis of biomass-derived polymers such as polyesters and polyamides, making it highly applicable in sectors like beverage bottles, textiles, food packaging, electronics, and automotive materials^26–31^.

The Life Cycle Assessment (LCA) method is a powerful tool that enables companies to identify potential environmental issues and optimize resource utilization by systematically evaluating the entire life cycle of a product, from raw material acquisition to final disposal, highlighting environmental impacts at every stage^24,32–36^. LCA has been widely applied to assess the production of high value-added chemical products from biomass. For instance, Jorge Blanco et al. (2020)^37^⁠ compares glucose production from maize starch (MS-bp) and woody biomass residues (WBR-bp), finding WBR-bp outperformed MS-bp in all six impact categories (e.g., 55% lower climate change potential: 0.8 vs. 1.76 kg CO₂ eq/kg glucose), with MS-bp’s starch production and WBR-bp’s pretreatment as key hotspots. Nurul Ain Abu-Bakar et al. (2023)⁠^38^⁠ assesses empty paddy grains (EPFG)-based glucose production across three scenarios: Scenario 1 has lower climate impact (1.08 kg CO₂ eq/kg) than other biomasses, Scenario 2 (lignin-powered electricity) reduces it by 29% and generates surplus electricity. Enzymatic hydrolysis is the main hotspot, with higher glucose yield mitigating impacts. Similarly, Essel et al. (2012)^24^ and Chen et al. (2016)^39^ compared the environmental performance of traditional petroleum-based bottles with bio-based alternatives, finding that biomass-based feedstocks offer notable advantages in reducing climate change impacts, fossil resource depletion, and raw material consumption, woody biomass, for example, reduced global warming potential (GWP) by 21% and required 22% less fossil fuel than fossil-based materials. However, bio-based products do not outperform fossil-based products across all environmental categories. Fagundes et al. (2024)^40^ reported that although bioethanol production from banana, potato, and papaya waste reduces fossil resource scarcity and greenhouse gas emissions from the uncontrolled decomposition of food waste, it may contribute more significantly to terrestrial ecotoxicity and marine eutrophication, mainly due to the use of phosphate buffer in the pretreatment stage, high energy consumption in the fermentation stage, and the production and consumption of enzymes in the enzymatic hydrolysis process. Therefore, a comprehensive evaluation of environmental benefits is essential to accurately assess the sustainability of bio-based products.

The development of feedstocks for HMF production can be broadly categorized into two phases: the first focused on monosaccharide-based feedstocks such as fructose and glucose, while the second shifted toward non-food biomass and agricultural wastes, including lignocellulose and bagasse^37^. Although monosaccharides were initially favored due to their high reactivity, their limited availability, high cost, and competition with food resources significantly restricted their potential for large-scale industrial application. In contrast, the second-generation feedstocks, non-food biomass and agricultural waste, have gained increasing attention due to their abundance, low cost, sustainability, and environmental benefits, positioning them as a key direction for future HMF production. For instance, HMF derived from mangosteen has demonstrated superior environmental performance compared to high-fructose corn syrup (HFCS) in several impact categories, including greenhouse gas emissions and ecotoxicity^41^. Similarly, LCA studies on HMF production from sago pith waste (SPW) have also been conducted^42^. The study uses a one-pot THF-water biphasic system, evaluates its environmental impacts via LCA, and compares it with the DMSO-water system⁠^42^. Under optimized conditions (160 °C, 45 min; aluminum sulfate + sodium chloride as catalysts), SPW achieves 32.6% HMF yield, with ~ 30% in the crude extract^42^⁠. LCA confirms the THF-water system’s significantly lower environmental impacts (higher HMF concentration), eliminating pre-fiber separation and simplifying processes^42^⁠. However, existing research still exhibits three major critical gaps: (1) Focusing on specific feedstocks like fruit waste and starch-based residues, it lacks LCA analysis for HMF production from typical crop residues such as straw, which boasts high yields and widespread distribution; (2) Environmental assessments are often confined to HMF as a standalone product, failing to extend to the full chain of “HMF oxidation for 2,5-furanedicarboxylic acid (FDCA) production,” thus unable to reflect the complete environmental footprint of high-value derivative manufacturing; (3) There has been no systematic comparison of environmental impacts between crop straw and traditional fructose feedstocks across the entire HMF-FDCA process, nor has there been an in-depth analysis of key environmental hotspots and optimization potential in straw-based processes.

To address the aforementioned research gaps, this study offers distinct novelty and unique contributions: (1) It is the first to conduct a full-chain LCA assessment of the “HMF production - oxidative conversion to FDCA” process using straw (a typical crop straw) as feedstock, filling a research void in this field for crop straws; (2) It simultaneously compares the environmental impacts of two FDCA production processes (different separation and purification routes after HMF oxidation), revealing the mechanisms by which process selection influences overall lifecycle environmental performance; (3) It systematically analyzes the differences in environmental hotspots between the straw-based process and the traditional fructose-based process across all lifecycle stages, identifying emission reduction potentials in key stages such as stover pretreatment and HMF oxidation. The findings provide comprehensive environmental sustainability references for high-value utilization of agricultural residues like crop straws and offer scientific basis for green optimization of the HMF-FDCA industrial chain. Material methods.

## HMF and FDCA production

### HMF production

**Fig. 2:**
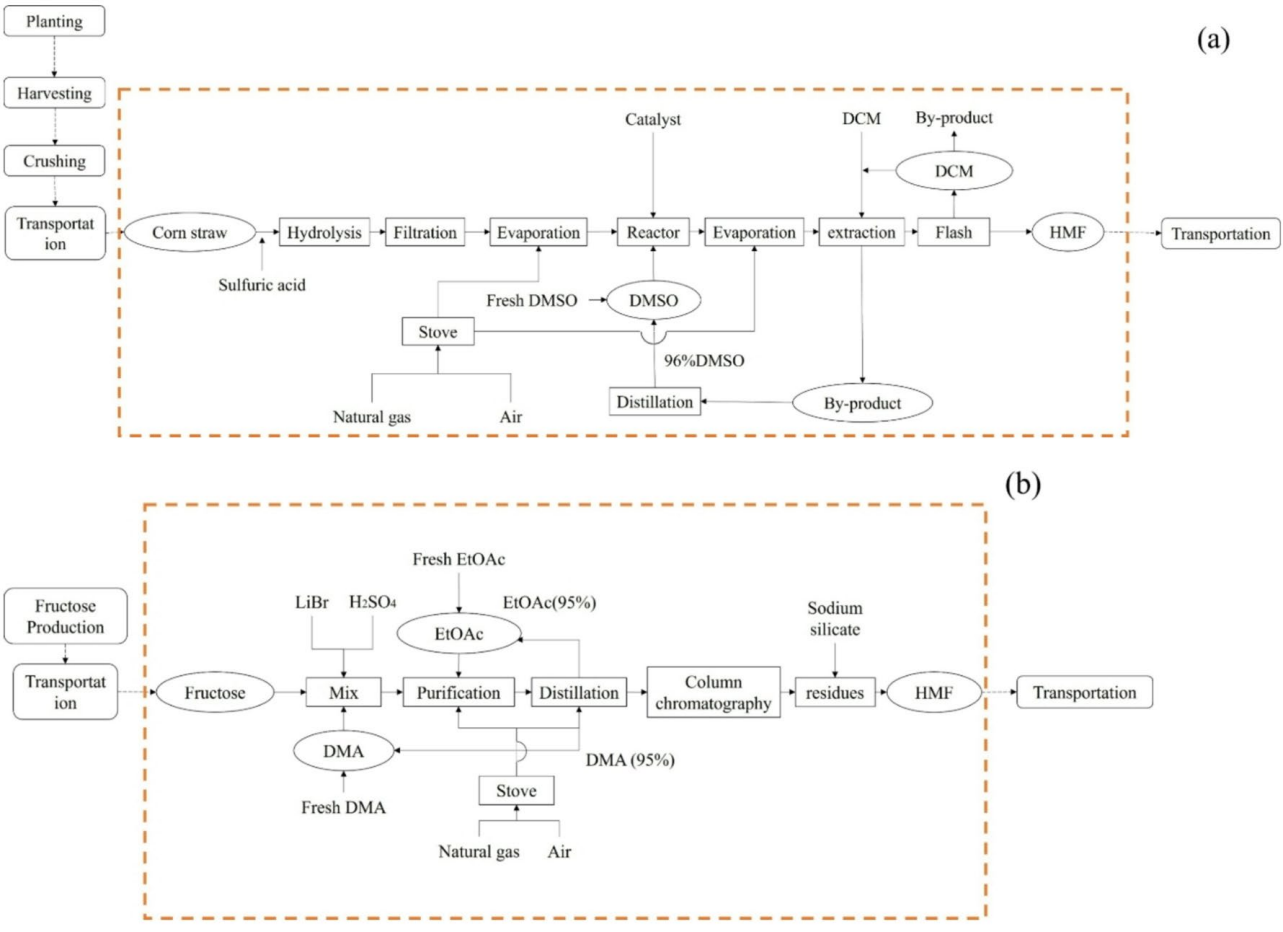
Scope of research on HMF production from straw **(a)**; HMF production from fructose **(b)**.

In this study, the HMF production process is divided into two main stages: HMF synthesis and HMF purification and recovery. The system boundary excludes the collection, crushing, and transportation of corn straw. As illustrated in Fig. 2(a) and Table 1, the production begins with the mixing of corn straw powder and dilute sulfuric acid, which is heated at 180 °C for a specified duration; the water is then evaporated to obtain a glucose-based precursor solution. This solution, along with a catalyst, is introduced into a reactor using a solvent mixture of dimethyl sulfoxide (DMSO) and water in a defined ratio. The reaction is conducted at 160 °C and 8.5 bar, yielding a liquid mixture rich in the target compound, HMF. The HMF-containing solution is subsequently purified using a liquid-liquid extraction column with dichloromethane (DCM) and water as the extraction solvents. The extracted mixture is processed through three flash separators operating at progressively lower pressures (1, 0.8, and 0.1 bar), resulting in HMF with a concentration of 97.11%. Both the DCM from the flash separators and the DMSO from the extraction column are collected and recycled to enhance process efficiency and reduce environmental impact.

**Table 1:** Key process Parameters, reaction Stoichiometry, and mass balance for HMF production from corn straw (Fig. 2a) and Fructose (Fig. 2b).

Process Type	Reaction Stoichiometry	Unit Operation	Operating Conditions	Conversion/Yield Factor	Mass Balance (based on feedstock, %)
Corn Straw-Based HMF (Fig. 2a)	1. (C_6_H_10_O_5_)n (Corn straw cellulose) + nH_2_O → nC_6_H_12_O_6_ (Glucose) (H_2_SO_4_-catalyzed) 2. C_6_H_12_O_6_ (Glucose) → C_6_H_6_O_3_ (HMF) + 3H_2_O (DMSO-water solvent, catalyst)	Hydrolysis	180 °C; dilute H_2_SO_4_	Corn straw → glucose yield: 70%	Corn straw + dilute H_2_SO_4_ (feed): 100% Hydrolysis effluent: 100% (contains glucose, residues)
		Filtration+ Evaporation	(filtration); pressure-reduced (evaporation)	Glucose retention rate: 95%	Filtrate (glucose solution): 66.5% Residues (discarded): 33.5%
		Reactor	160 °C; 8.5 bar, DMSO-water solvent	Glucose → HMF conversion: 85%	Reactor effluent (HMF-rich mixture): 66.5% (glucose solution feed)
		Evaporation+ Extraction	Pressure-reduced (evaporation); DCM-water extraction	HMF extraction rate: 98%	Extracted HMF-DCM mixture: 56.5% DMSO (recycled): 96% (from extraction)
		Flash Separators	1 bar → 0.8 bar → 0.1 bar (progressive)	HMF concentration rate: 97.11%	Final HMF (output): 54.9% DCM (recycled): 95% (from separators)
Fructose-Based HMF (Fig. 2b)	C_6_H_12_O_6_ (Fructose) → C_6_H_6_O_3_ (HMF) + 3H_2_O (LiBr + H_2_SO_4_-catalyzed, DMA solvent)	Mixing	(fructose + DMA + LiBr + H_2_SO_4_)	Mixing uniformity: 100% (homogeneous)	Fructose + DMA + catalysts (feed): 100% Mixed solution: 100%
		Reaction	100 °C, 6 h	Fructose → HMF yield: 45%	Reaction effluent (HMF mixture): 100%
		Purification+ Evaporation	EtOAc + sodium silicate (purification); evaporator	HMF retention rate: 90%	Purified solution: 45% DMA (recycled): 95%
		Chromatography	Ethyl acetate-hexane, 2.5 h	HMF purification rate: 98%	Chromatographed HMF solution: 44.1%
		Drying	Anhydrous sodium sulfate	HMF drying yield: 99%	Final HMF (output): 43.7%

The production process of HMF from fructose is illustrated in Fig. 2(b) and Table 1. Initially, a fructose solution is thoroughly mixed with N, N-dimethylacetamide (DMA), followed by the addition of lithium bromide (LiBr) and sulfuric acid (H_2_SO₄). The mixture is then heated to 100 °C and maintained at that temperature for 6 h, resulting in a liquid containing the target product, HMF. This mixture undergoes purification using ethyl acetate (EtOAc) and sodium silicate, and is processed with a rotary evaporator (1300 W, 5 min) under reduced pressure to remove DMA. Subsequently, the solution is further purified via ethyl acetate-hexane chromatography for 2.5 h. Finally, the remaining product is dried with anhydrous sodium sulfate to obtain the final HMF product. This method achieves a 45% yield in converting fructose to HMF.

### FDCA production

**Fig. 3:**
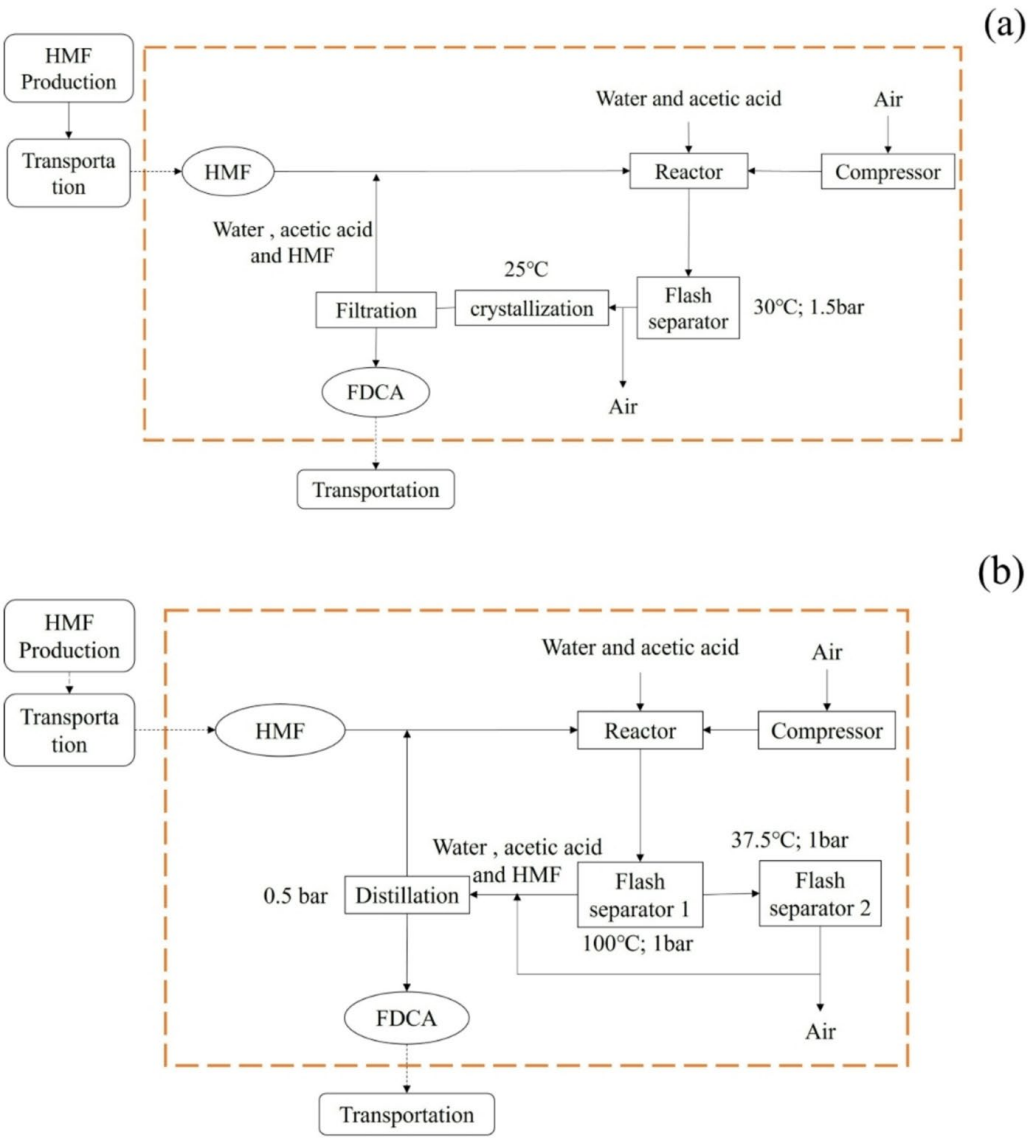
FDCA from HMF oxidation, crystallization **(a)** and distillation **(b)**.

In this study, the production process of FDCA from HMF is depicted in Fig. 3(a) and Table 2 is divided into three main stages: FDCA synthesis, FDCA isolation, and FDCA purification and recovery. The system boundary excludes the production and transportation of HMF. The crystallization-based FDCA production process begins with the addition of HMF, acetic acid, a Pt/ZrO_2_ catalyst, and compressed air into a reactor, operating at a temperature of 100 °C and a pressure of 10 bar. Following the reaction, an initial separation is performed using a flash separator set at 30 °C and 1.5 bar. The resulting stream is then directed into a crystallization unit maintained at 25 °C, where FDCA crystallizes out. Finally, the solid FDCA is collected using a rotary vacuum filter, while the remaining liquid is recycled back into the reactor feed, enhancing the efficiency and sustainability of the process.

**Table 2:** Reaction Stoichiometry, operating Parameters, and mass balance for Biomass-Derived FDCA production from HMF: crystallization and distillation Processes.

Process Type	Reaction Stoichiometry	Unit Operation	Operating Conditions	Conversion/Yield Factor	Mass Balance (based on HMF feed, %)
Crystallization Process (Fig. 3a)	C_6_H_6_O_3_ (HMF) + 2O_2_ → C_6_H_4_O_5_ (FDCA) + 2H_2_O (Pt/ZrO_2_-catalyzed)	Reactor	100 °C; 10 bar	HMF conversion: 99.7%	HMF + acetic acid + water + air (feed): 100% Reactor effluent (contains FDCA, trace unreacted HMF, solvent): 100%
		Flash Separator	30 °C; 1.5 bar	Air separation rate: 99%	Air (discharged): 4.95% (99% of feed air; feed air accounts for ~ 5% of total feed) Liquid phase (to crystallization): 95.05% (contains FDCA, solvent, trace unreacted HMF)
		Crystallization Unit	25 °C	FDCA crystallization rate: 99.9%	FDCA crystals (to filtration): 99.6% Mother liquor (recycled): 0.25% (contains trace uncrystallized FDCA, solvent)
		Filter	-	FDCA filtration yield: 99.9%	FDCA product (output): 99.5% Filtrate (recycled to reactor): 0.25% + 0.001% (trace filtration loss)
Distillation Process (Fig. 3b)	Same as crystallization process reaction equation	Reactor	100 °C, 10 bar	HMF conversion: 99.7%	HMF + acetic acid + water + air (feed): 100% Reactor effluent: 100%
		Flash Separator 1	100 °C, 1 bar	Gas-liquid separation rate: 30% (gas phase proportion)	Gas phase (to Flash Separator 2): 30% (contains air, water, acetic acid) Liquid phase (to distillation): 70% (contains FDCA, solvent)
		Flash Separator 2	37.5 °C, 1 bar	Air separation rate: 99.5%	Air (discharged): 5.97% Water + acetic acid (recycled): 24.03%
		Distillation Column	0.5 bar	FDCA distillation purity: 99.9%	FDCA product (output): 99.6% Residue (recycled): 0.1% (contains trace FDCA, solvent)

The FDCA production process via distillation is illustrated in Fig. 3(b) and Table 2. Initially, HMF, acetic acid, a Pt/ZrO_2_ catalyst, and compressed air are introduced into a reactor operating at 100 °C and 10 bar. Following the reaction, air, water, and acetic acid are separated in a flash separator set at 100 °C and 1 bar. The vapor stream from this separator is directed into a second flash separator maintained at 37.5 °C and 1 bar, where air is removed from the gas mixture, allowing water and acetic acid to be recirculated back into the reactor feed. Meanwhile, the liquid-phase output from the first flash separator undergoes final product purification in a distillation column operating at 0.5 bar. This process achieves a high conversion efficiency, yielding FDCA from HMF at 99.7%.

### LCA

The LCA in this study is conducted in accordance with ISO 14040-44 standards (ISO 14040:2006; ISO 14044:2006) and is structured into three main phases: goal and scope definition, inventory analysis, and impact assessment. The analysis encompasses both HMF and FDCA production processes, evaluating their environmental impacts throughout the defined system boundaries.

### Goal and scope definition

The LCA in this study is conducted in accordance with ISO 14040-44 standards (ISO 14040:2006; ISO 14044:2006) and is structured into three main phases: goal and scope definition, inventory analysis, and impact assessment. The analysis encompasses both HMF and FDCA production processes, evaluating their environmental impacts throughout the defined system boundaries.

### Functional unit

The functional unit in LCA quantitatively defines the product or process being analyzed, serving as a reference point for data collection and enabling consistent, comparable evaluations of environmental impacts across different products or processes. In this study, 1 ton of corn straw is selected as the functional unit for the production of HMF, while 284.34 kg of HMF is used as the functional unit for the subsequent production of FDCA. These functional units provide a standardized basis for assessing and comparing the environmental performance of each stage in the biomass conversion process.

### System boundaries

Appropriate definition of the system boundaries are essential to ensure the comprehensiveness and accuracy of LCA, as they prevent one-sided or exaggerated evaluations of environmental impacts. The system boundaries act as a conceptual “fence” that includes all processes and activities directly relevant to the study’s objectives, while excluding those deemed minor or irrelevant, thereby making the analysis more focused and manageable. In this study, the system boundaries encompass the production of both HMF and FDCA. These systems involve the use of solvents, catalysts, energy, and various operational equipment. Two distinct production methods are used for HMF, each involving different solvents, catalysts, energy consumption patterns, and equipment. A detailed breakdown of these components is presented in the inventory analysis. For FDCA production, the two compared systems, crystallization and distillation, differ primarily in the equipment used for product separation and purification. Specifically, the crystallization method utilizes a flash separator, while the distillation method employs two flash separators at different stages. However, both methods use similar solvents and catalysts. It is important to note that the scope of this study includes only the synthesis, separation, and purification of the target products (HMF and FDCA). Activities such as raw material transportation, pretreatment, final product distribution, the treatment of the wastewater and solid waste are excluded from the system boundary. These defines processes collectively establish the framework for the LCA conducted in this study.

To facilitate comparison and ensure consistency in the Life Cycle Assessment, the following assumptions are made in this study: (1) The electrical composition of this scenario is based on China’s current actual energy structure (62% fossil fuels, 40% non-fossil fuels), set at 60% coal power + 40% renewable energy (where renewable energy includes hydropower, wind power, and photovoltaic, accounting for 45%, 30%, and 20% respectively, consistent with China’s actual installed capacity structure for renewable energy). (2) Corn straw is primarily composed of hemicellulose, cellulose, and lignin; however, only cellulose is considered as a viable precursor for HMF synthesis in this study, as hemicellulose and lignin are not suitable for this purpose. (3) It is assumed that there are no liquid or gas leakages from production equipment during the synthesis of HMF and FDCA, thereby ensuring process stability and minimizing unintended environmental emissions.

### Inventory analysis

Inventory analysis involves the detailed quantification of all material and energy inputs and outputs associated with each stage of a product’s life cycle. In this study, data for each process were collected through questionnaires and interviews with the organizations responsible for developing the technologies listed in Tables 3, 4, 5 and 6. This approach ensures that the inventory data are based on practical, real-world operational conditions, enhancing the accuracy and reliability of the Life Cycle Assessment.

**Table 3:** Input-Output table for corn Straw-Based HMF (HMF_Straw_) production Stages.

Stage	Category	Material/Product Item	Unit	Value
**Synthesis**	Input	Corn straw	kg	1000
		Water	kg	2642.84
		Sulfuric acid	kg	68.63
		DMSO	kg	26.81
		Al₂O₃	kg	6.58
		Electricity	kWh	247.36
	Output	HMF-containing mixture	-	Generated
**Purification and recovery**	Input	DCM	kg	42.57
		Electricity	kWh	989.43
	Output	HMF	kg	284.34
		Wastewater	m³	6874.64
		Solid waste	kg	87.52
	Recycled item	DCM; DMSO	-	Recycled for reuse

**Table 4:** Input-Output table for Fructose-Based HMF (HMF_Fructose_) production Stages.

Stage	Category	Material/Product Item	Unit	Value
**Synthesis**	Input	Fructose	kg	770.37
		Water	kg	1124.84
		Sulfuric acid	kg	25.42
		LiBr	kg	38.52
		DMA	kg	65.41
		Electricity	kWh	344.66
	Output	HMF-containing mixture	-	Generated
**Purification and recovery**	Input	EtOAc	kg	23.11
		Sodium silicate	kg	24.72
		Electricity	kWh	1033.96
	Output	HMF	kg	284.34
		Wastewater	m³	7759.47
		Solid waste	kg	114.83
	Recycled item	DMA	-	Recycled for reuse

**Table 5:** Input-Output table for Crystallization-Based FDCA (FDCA_crystallization_) production Stages.

Stage	Category	Material/Product Item	Unit	Value
**Synthesis**	Input	HMF	kg	284.34
		Water	kg	1147.92
		Acetic acid	kg	124.67
		Pt-ZrO₂	kg	13.54
		Electricity	kWh	12.49
		Heating	kWh	143.63
		Compressed air	-	Introduced
	Output	Reaction mixture	-	Received
**Separation**	Input	Electricity	kWh	99.94
		Heating	kWh	1149.01
		Separated stream	-	Generated
	Output	Separated stream	-	Received
**Purification and recovery**	Input	Separated stream	-	Received
		Electricity	kWh	12.49
		Heating	kWh	143.63
	Output	FDCA	kg	295.64
		Wastewater	m³	1627.38
		Solid waste	kg	114.83
		Nitrogen	kg	87.69
		Oxygen	kg	22.59
	Recycled item	Remaining liquid	-	Recycled for reuse

**Table 6:** Input-Output table for Distillation-Based FDCA (FDCA_distillation_) production Stage.

Stage	Category	Material/Product Item	Unit	Value
**Synthesis**	Input	HMF	kg	284.34
		Pt-ZrO₂	kg	14.27
		Electricity	kWh	12.20
		Heating	kWh	174.56
		Compressed air	-	Introduced
	Output	Reaction mixture	-	Generated
**Separation**	Input	Reaction mixture	-	Received
		Electricity	kWh	24.39
		Heating	kWh	349.12
	Output	Separated air	-	Discharged
		Water & acetic acid	-	Recycled for reuse
		Liquid stream	-	Generated
**Purification and recovery**	Input	Liquid stream	-	Received
		Electricity	kWh	85.36
		Heating	kWh	1221.93
	Output	FDCA	kg	295.64
		Wastewater	m³	1789.24
		Solid waste	kg	87.52
		Nitrogen	kg	92.67
		Oxygen	kg	23.87

### Impact assessment

Impact assessment plays a crucial role in identifying the key processes and factors that exert the most significant environmental influence throughout a product’s life cycle. In this study, the LCA is performed using OpenLCA (2.0.4) software, employing the CML 1 A impact assessment method developed by Leiden University in the Netherlands. CML 1 A follows a “midpoint-problem-oriented” approach, utilizing 11 well-established, publicly available, and transparent indicators, such as kg CO_2_-eq and kg SO_2_-eq, to generate life cycle impact results that align with international standards. The method is widely recognized in the industry for its simplicity, flexibility, scalability, and minimal data requirements, eliminating the need for complex damage modeling. It has been successfully applied in several prior studies^35,36,43–45^. In this study, 11 impact categories are selected for evaluation (Table 7), including: Abiotic Depletion (AD), Acidification (AC), Eutrophication (EU) (evaluated only for HMF production), Freshwater Aquatic Ecotoxicity 100a (FAE), Freshwater Sediment Ecotoxicity 100a (FSE), Global Warming Potential 100a (GWP), Human Toxicity 100a (HT), Marine Aquatic Ecotoxicity 100a (MAE), Marine Sediment Ecotoxicity 100a (MSE), Photochemical Oxidation (PO), and Terrestrial Ecotoxicity 100a (TE) (evaluated only for FDCA production).

**Table 7:** CML1A analysis method impact categories.

Impact category	Abbreviation	Unit
Abiotic depletion	AD	kg Sb eq
Acidification	AC	kg SO_2_ eq
Eutrophication	EU	kg PO_4_^−^ eq
Freshwater aquatic ecotox. 100a	FAE	kg 1,4-DB eq
Freshwater sediment ecotox. 100a	FSE	kg 1,4-DB eq
Global warming potential 100a	GWP	kg CO_2_ eq
Human toxicity 100a	HT	kg 1,4-DB eq
Marine aquatic ecotox. 100a	MAE	kg 1,4-DB eq
Marine sediment ecotox. 100a	MSE	kg 1,4-DB eq
Photochemical oxidation	PO	kg C_2_H_4_ eq
Terrestrial ecotoxicity 100a	TE	kg 1,4-DB eq

The life cycle assessment (LCA) data supporting this study is primarily constructed using the Tiangong Life Cycle Assessment Database (v 0.2.0). This database serves as a localized LCA core repository tailored to China’s industrial production scenarios. Its data comprehensively covers critical lifecycle stages including energy consumption, material production, and pollutant emissions. Having undergone systematic localization validation and calibration, it precisely meets the assessment requirements for processes such as “straw/fructose-derived HMF” and “crystallization/distillation-derived FDCA” examined in this study.

## Discussion of results

### HMF production

**Fig. 4:**
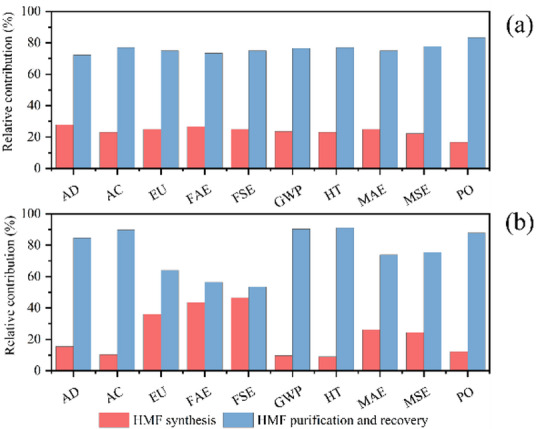
Relative contribution of stages in HMF production, fructose **(a)**; straw **(b)**.

Figure 4 summarizes the results of HMF production using 1 ton of corn straw as feedstock and compares them with the conventional HMF production process using fructose, across all stages, namely, HMF synthesis and HMF purification and recovery. In the visual representation, the x-axis denotes the environmental impact categories, while the y-axis shows the percentage contribution relative to the highest impact observed in each category, normalized to 100%. In the case of HMF production from fructose (Fig. 4(a)), the purification and recovery stage contributes the most to all environmental impact categories, with each contributing more than 70%. Specifically, acidification (AC), freshwater aquatic ecotoxicity (FAE), global warming potential (GWP), and human toxicity (HT) exhibit contributions of 76.92%, 73.33%, 76.47%, and 76.92%, respectively. This result is consistent with the findings of Goetz’s on the production of HMF from fructose-based HFCS^41^. These findings suggest that, for future environmental optimization, the purification and recovery processes should be prioritized- specifically by adopting an integrated “high-efficiency separation + closed-loop solvent recovery” process to improve the regeneration efficiency of extractants such as ethyl acetate to over 95%, reducing ecotoxicity and resource consumption from solvent loss. In the corn straw-based HMF production process (Fig. 4(b)), contributions to FAE emissions from product synthesis and purification/recovery are relatively balanced, accounting for 43.58% and 56.42%, respectively. This indicates the absence of a single dominant contributor, implying that emission reductions will require a combined or systematic approach: improving cellulose conversion and reducing catalyst dosage in synthesis. In contrast, for AC, GWP, and HT categories, the purification and recovery stage is the dominant contributor, with values of 89.74%, 90.32%, and 91.07%, respectively, while the product synthesis stage accounts for only 10.26%, 9.68%, and 8.93%, respectively. These results highlight that purification and recovery are the primary contributors to environmental impacts in these categories and should be the key focus of subsequent system optimization efforts. Consistent with findings from studies such as Vioni’s finding on HMF production from sorghum pulp waste (SPW), the purification stage emerged as the primary contributor to GWP and toxicity indicators regardless of the feedstock—whether agricultural waste (straw) or SPW^42^. However, SPW exhibited higher overall environmental impacts than straw due to the laboratory scale (1 g feedstock) and incomplete solvent recovery (20%).

**Fig. 5:**
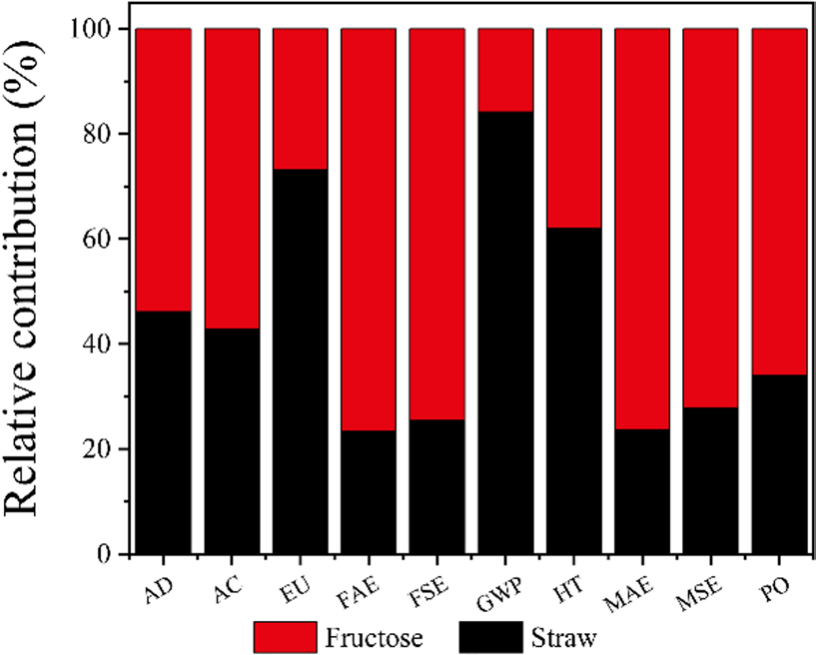
Relative contribution of HMF production by fructose vs. straw as feedstock.

**Fig. 6:**
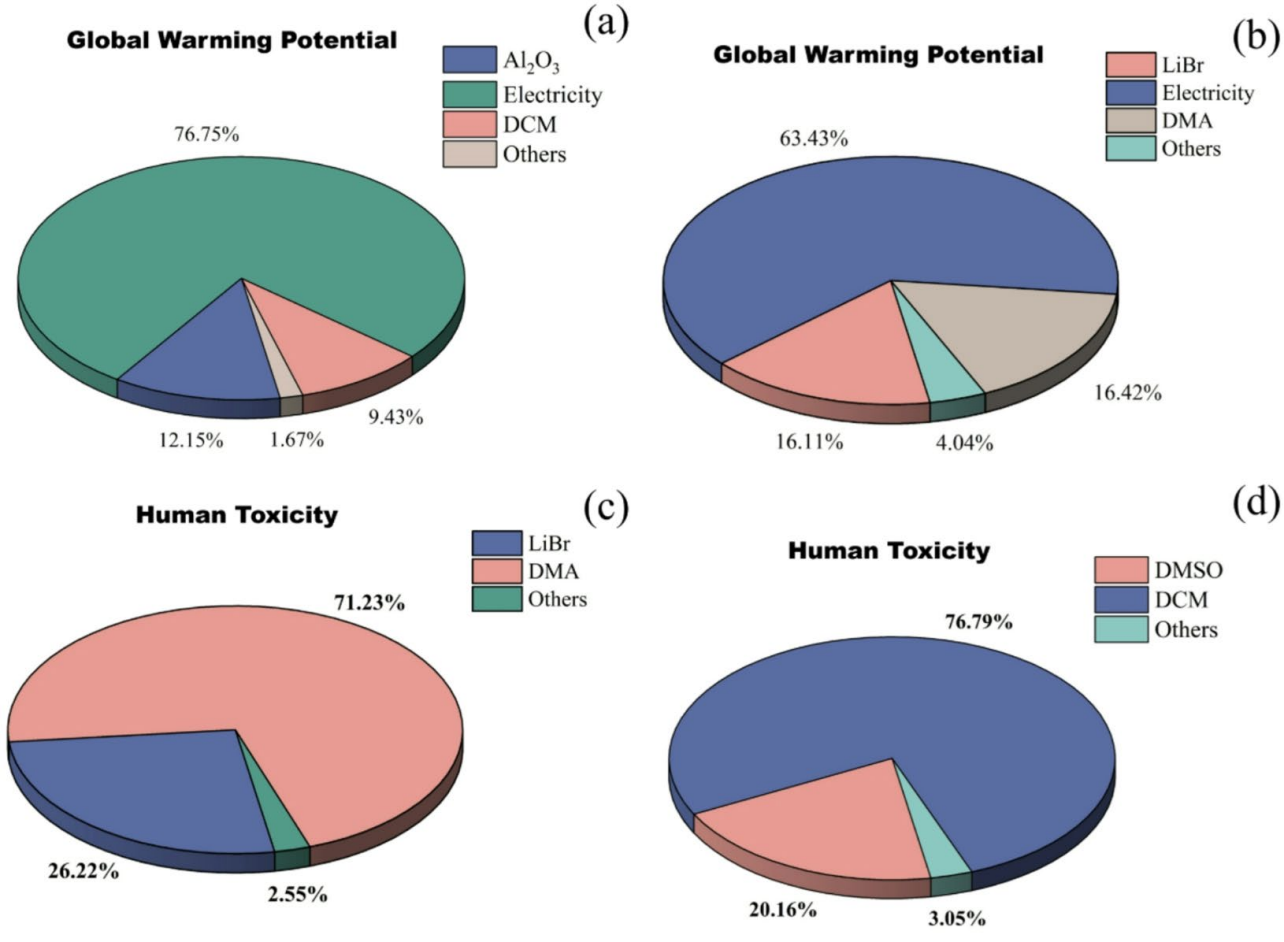
The global warming potential contributions of the entire process from **(a)** straw and **(b)** fructose. The human toxicity contributions of the entire process from **(c)** fructose and **(d)** straw.

Figure 5 presents the overall relative contributions of HMF production from fructose and corn straw across various environmental impact categories. Fructose-based HMF production exhibits greater environmental impacts than straw-based production in all categories, with the most significant differences observed in freshwater aquatic ecotoxicity (FAE), freshwater sediment ecotoxicity (FSE), marine aquatic ecotoxicity (MAE), and marine sediment ecotoxicity (MSE), where the relative contributions reach 23.46%, 25.48%, 23.58%, and 27.83%, respectively. These elevated impacts are likely associated with the use of lithium bromide (LiBr) in the fructose-based synthesis process. In freshwater ecosystems, high concentrations of bromide ions (Br⁻) can directly damage fish gill tissues, disrupt osmoregulation in crustaceans, and lead to genetic toxicity through the formation of disinfection byproducts such as bromoform. In freshwater sediments, Br⁻ binds to iron and aluminum oxides, causing soil compaction and inhibiting the activity of nitrifying bacteria, which disrupts the nitrogen cycle. In marine environments, Br⁻ may form ozone-depleting methyl bromide (CH₃Br) and genotoxic organic bromides (e.g., total organic bromine compounds, TOBr), which interfere with the development of calcifying marine organisms. Although Br⁻ does not directly participate in reactions in marine sediments, high salinity may enhance the activity of sulfate-reducing bacteria under anoxic conditions, catalyzing the production of toxic hydrogen sulfide (H_2_S), which is harmful to benthic life. Additionally, soluble organic matter can bind with Br⁻ to form a wide range of unidentified brominated byproducts, comprising approximately 60%−80% of the total, posing long-term ecological risks. Only three impact categories, eutrophication (EU), global warming potential (GWP), and human toxicity (HT), showed values exceeding 50%, with contributions of 73.24%, 84.19%, and 61.99%, respectively. Notably, this trend aligns with the general advantages of second-generation biomass feedstocks: Goetz et al. (2023)^41^compared corn-based (first-generation) and miscanthus-based (second-generation) HMF production, finding that miscanthus-based GWP (2.23 kg CO₂ eq/kg HMF) was lower than corn-based (2.64 kg CO₂ eq/kg HMF), with superior environmental metrics across all indicators except land use. The GWP reduction trend observed in glucose production from woody biomass residues (WBR) compared to corn starch (0.8 kg CO₂ eq/kg vs. 1.76 kg CO₂ eq/kg) aligns with the findings^37^, collectively demonstrating the potential of non-food lignocellulosic feedstocks for greenhouse gas mitigation. Using straw as raw material results in 87.73 kg CO_2_ eq less emissions than using fructose, when producing the same quality of HMF. The variation in global warming potential (GWP) is primarily attributed to the consumption of electricity in both processes (Fig. 6(a, b)). Numerous studies have established a strong correlation between fossil fuel usage and global warming potential, as the combustion of these fuels emits greenhouse gases (GHGs), which are significant contributors to climate change and have initiated a range of complex environmental and climatic impacts^46–48^. At the same time, 7.87 kg 1,4-DB eq is reduced in emissions from the straw production HMF system. Human toxicity (HT), as assessed in LCA, refers to the potential adverse effects on human health caused by chemicals or pollutants emitted during the entire life cycle of a product, service, or process, from raw material extraction and production to usage and disposal. This potential is quantified using specialized toxicity assessment models and relevant toxicological data, encompassing harmful substances such as heavy metals, organic compounds, and air pollutants. DMA is the primary contributor to human toxicity (HT) in the production of HMF from fructose (Fig. 6(c)). Previous studies have shown that DMA is classified as a moderately toxic solvent, which poses certain hazards to human health. It is primarily metabolized by the liver, potentially causing elevated transaminase levels and jaundice. Inhalation of high-concentration DMA vapors may suppress the central nervous system, leading to headaches and unconsciousness. Direct contact can irritate the skin and eyes^49,50^. Additionally, the use of dichloromethane (DCM) significantly contributes to HT in straw-based HMF production (Fig. 6(d)), as prior studies have demonstrated that DCM is quickly absorbed and widely distributed in the human body^51,52^. However, subsequent research suggests that DCM can be recovered and purified for reuse as an extractant, and it may be substituted with less toxic chemical agents that offer similar functional benefits^53^. One such promising alternative is γ-valerolactone (GVL), a biomass-derived compound that offers enhanced sustainability and lower toxicity^54–57^.

In addition, Table 8 systematically presents the key environmental impact results of various feedstock pathways under the LCA framework, with all indicators normalized to the production of 100 kg of HMF. Since the methods used in this study differ from those used in the referenced studies, only the impact categories that overlap with the CML 1 A method used in this study are listed. Despite the disparities in research approaches, the impact categories in question rely on analogous calculation models and measurement procedures. This congruence in methodological underpinnings culminates in correspondingly similar final calculation outcomes, such as Global Warming Potential (GWP) (GWP as per IPCC 2013 guidelines); Acidification (AC harnessed through the RAINS model); and Eutrophication (EU anchored in the framework established by Heijungs in 1992). Among the examined feedstocks, fructose and HFCS are classified as first-generation sources, whereas straw, miscanthus, and SPW fall under second-generation feedstocks. The GWP of straw is 164.48 kg CO₂ eq/100 kg HMF, lower than fructose’s 195.37 kg CO₂ eq; HFCS’s GWP reaches 312.00 kg CO₂ eq, significantly higher than miscanthus’s 223.00 kg CO₂ eq. The environmental impact of producing HMF using second-generation raw materials is significantly lower than that of first-generation raw materials (fructose and straw; HFCS and miscanthus). This indicates that producing HMF using second-generation raw materials is a more environmentally friendly option compared to first-generation raw materials. Notably, straw and SPW are considered agricultural residues, byproducts generated during routine agricultural operations, while miscanthus is categorized as a “herbaceous energy crop,” specifically cultivated for biofuel or industrial applications and not as a byproduct of primary agricultural activities. Life cycle results indicate that HMF production using straw as a raw material exhibits a lower environmental impact across several key indicators, including global warming potential (GWP), eutrophication (EU), fossil abiotic energy (FAE), and human toxicity (HT), compared to production using miscanthus. This is because cultivating miscanthus requires dedicated arable land, with its land use change (LUC) contributing 15%–25% to the global warming potential (GWP)^41^. In contrast, straw, an agricultural byproduct, requires no additional land, and its indirect land use change (ILUC) has a negligible impact on the total GWP^40^. This represents a key structural factor underpinning straw’s superior GWP. This suggests that utilizing straw, an agricultural waste product, is more environmentally sustainable than using cultivated energy crops such as miscanthus. The acidification (AC) of HMF produced from straw as a raw material is significantly higher than that of miscanthus, which is related to the use of KOH in the lignin depolymerization process in the HMF production system of miscanthus. However, the use of SPW as a feedstock results in a markedly higher environmental impact than both straw and miscanthus. It is noteworthy that the environmental impact of producing HMF from SPW as feedstock is significantly greater than that from the other two feedstocks (straw and Miscanthus). This is because the functional unit in that study was the consumption of 1 g of SPW, which differs substantially from the functional unit in this study (1 t of straw). Furthermore, the laboratory-scale nature of that study imposes certain limitations (e.g., an incomplete solvent recovery rate of 20%). Scaling the process to an industrial level would necessitate redefining system boundaries and parameters, which could alter impact intensity due to scale effects. Verdnik et al. (2022)^59^ find that when scaling up processes from laboratory scale (grams) to industrial scale (tons), employing continuous-flow microreactor designs and process intensification technologies can reduce energy consumption by 15–22% and CO₂ emissions by 12–48%. In industrial-scale production, Verdnik et al. (2022)^59^ enhance product selectivity from 75% in the lab to over 95% by optimizing microchannel mixing structures and in-situ separation/recovery techniques, reducing byproduct-related environmental impacts by approximately 82%. Referencing the scaling-up principles of microreactors, the proportion of thermal losses in the reaction system will decrease from 40% at the laboratory scale to below 22% at the industrial scale. Under equivalent experimental conditions, where 1,000 kg of straw or miscanthus are used as input material, the environmental advantages of straw become clearly evident, offering a promising theoretical foundation for the future large-scale production of HMF from agricultural waste.

**Table 8:** Comparison with other HMF production LCAs, per 100 kg of HMF produced.

Impact category	GWP	AC	EU	FAE	HT	reference
	kg CO_2_ eq	kg SO_2_ eq	kg PO_4_^−^ eq	kg 1,4-DB eq	kg 1,4-DB eq	
Straw	164.48	5.92	0.064	0.88	6.13	This study
Fructose	195.37	13.81	0.092	3.74	7.28	This study
HFCS	312.00	1.99	0.37	-	-	^41^
Miscanthus	223.00	1.60	0.11	-	-	^41^
SPW	644.18	3.10	0	30.68	1046.02	^42^

**Fig. 7:**
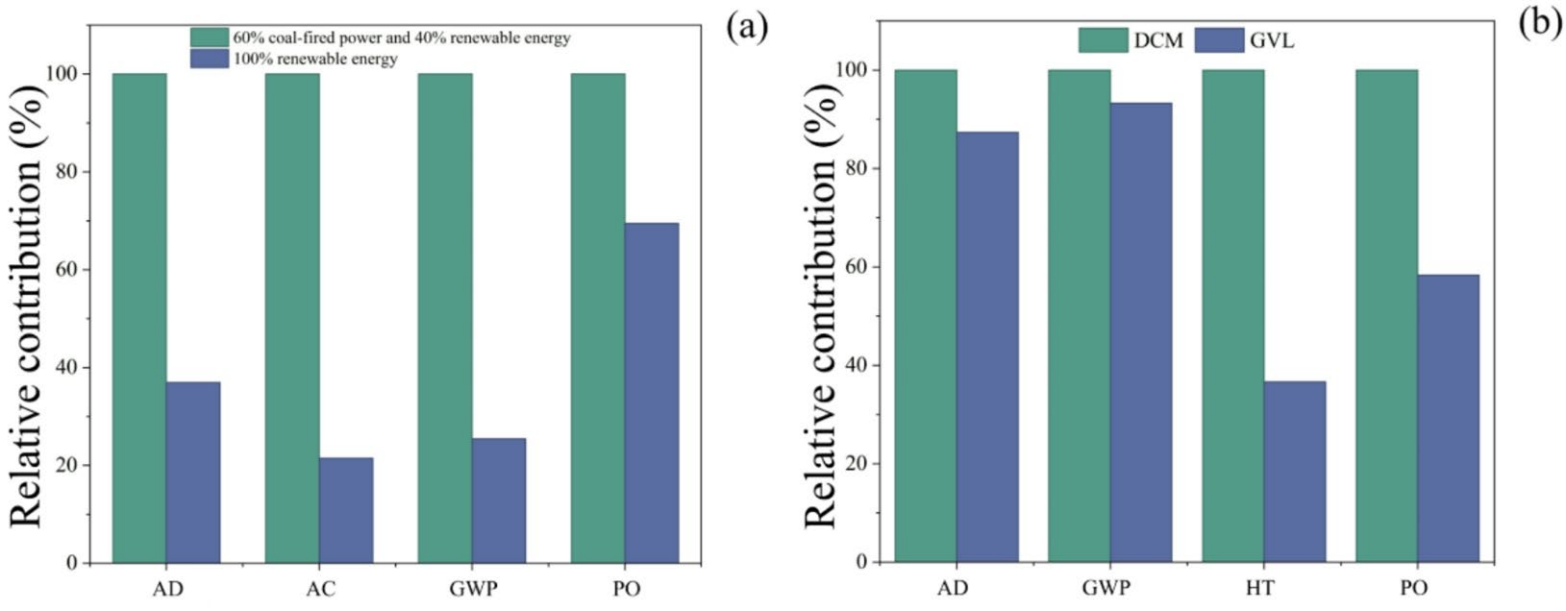
Sensitivity analysis evaluating use of electricity and solvent alternatives of HMF_straw_; charts compare 60% coal-fired power + 40% renewable energy and 100% renewable energy **(a)**; DCM and GVL **(b)**.

To validate the robustness and reliability of the LCA results, a sensitivity analysis is conducted for HMF production from straw. It identifies the key parameters that significantly influence the evaluation outcomes, which clarifies the quantitative effects of parameter fluctuations on environmental impact potential, mitigates conclusion biases caused by data uncertainties and assumption deviations and provides a scientific basis for LCA decision-making applications. As shown in Fig. 6(a), electricity is the primary contributor to the GWP of straw-based HMF production, accounting for 76.75%—significantly higher than other stages such as Al₂O₃ (12.15%) and DCM (1.67%). This proportion directly validates the dominant role of the power structure in environmental impacts. The baseline power scenario in this study references China’s actual energy mix, set as “60% coal power + 40% renewable energy” (where renewable energy comprises 45% hydropower, 30% wind power, 20% photovoltaic power, and 5% biomass power). Simultaneously, based on the power generation development direction aligned with the “dual carbon” goals, an alternative scenario of “100% renewable energy” is established. As shown in the relative contribution comparison diagram in Fig. 7(a), under the 100% renewable energy scenario, the relative contribution of GWP decreases significantly compared to the baseline scenario (corresponding to a 74.56% reduction in emissions). Simultaneously, the relative contributions of non-biological resource consumption (AD), acidification (AC), and photochemical oxidation (PO) also decrease by 63.06%, 78.48%, and 30.52%, respectively. The core logic behind this emissions reduction lies in the fact that the lifecycle carbon emission factors of renewable energy sources (hydro, wind, and solar photovoltaic) are only 5%−10% of those from coal-fired power. This approach eliminates CO₂ emissions from coal combustion (GWP), reduces acid gas emissions such as SO₂ and NOₓ (AC), and simultaneously lowers dependence on fossil resources (AD). Currently, while China’s levelized cost of wind/solar power has declined by 60–80% since 2010, achieving 100% renewable energy supply for industrial load customers will require behind-the-meter storage or certified green power purchase agreements to cover approximately 25% of residual load hours when the grid’s renewable energy share drops below 30%. Recent pilot PPA prices ($0.07–0.09/kWh) are already 10% below the industrial average electricity rate, demonstrating economic viability under existing market mechanisms. However, hourly balancing costs ($0.01–0.015/kWh) must be incorporated into future economic life cycle assessment (LCA). As shown in Fig. 6(d), DCM is the primary contributor to HT in straw-based HMF production, accounting for a significant 76.79% of the total - substantially higher than other steps such as DMSO (20.16%). This proportion highlights the critical influence of solvent type on human toxicity. γ-valerolactone (GVL), a C₅ cyclic ester derived from lignocellulose, possesses the characteristics of being “green, non-toxic, and fully biodegradable.” GVL can directly replace DCM, enabling the establishment of a sensitivity scenario where GVL substitutes DCM. As shown in the relative contribution comparison in Fig. 7(b), after GVL substitution, the relative contribution of HT is significantly reduced compared to the DCM scenario (corresponding to a 63.36% reduction in emissions). Simultaneously, AD, GWP, and PO decrease by 12.68%, 6.72%, and 41.68%, respectively. This improvement stems not only from GVL’s low toxicity (its metabolism follows the tricarboxylic acid cycle without accumulation of harmful intermediate products). Additionally, because it uses biomass as feedstock, it reduces the consumption of petroleum-based resources required for DCM production (AD). Simultaneously, volatile organic compound emissions are reduced (PO). The sensitivity analysis results clearly identify the direction for environmental optimization in straw-based HMF production: prioritizing the adoption of 100% renewable energy for power supply and replacing DCM with GVL as the solvent can significantly mitigate core environmental impacts such as GWP and HT. This provides a concrete technical pathway for ensuring the environmental sustainability of its industrial-scale production.

### FDCA production

**Fig. 8:**
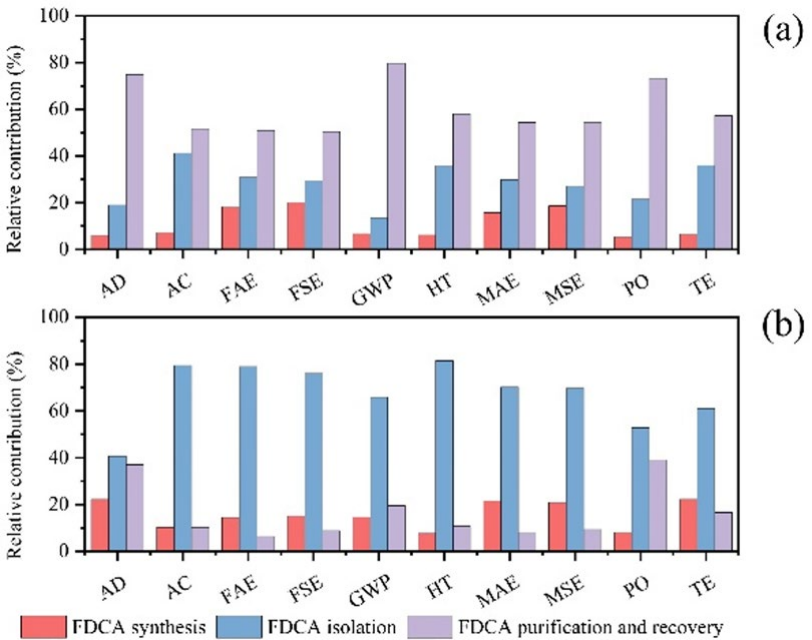
Relative contribution of stages in FDCA production, distillation **(a)**; crystallization **(b)**.

Figure 8 presents a comparative analysis of the environmental impact associated with various stages of FDCA production, including synthesis, separation, and purification/recovery, via two distinct methods: distillation (a) and crystallization (b). In the distillation route (Fig. 8(a)), the ranking of environmental impact contributions follows the order: product purification and recovery > product separation > product synthesis. Specifically, product purification and recovery account for the highest contribution to global warming potential (GWP) at 79.66%, whereas product synthesis and separation contribute 6.78% and 13.56%, respectively. This is likely due to the relatively high energy demand associated with the purification and recovery stage, particularly under vacuum conditions (0.5 bar), which increases electricity consumption and thus carbon emissions. These findings underscore that product purification and recovery represent critical stages for emission reduction in distillation-based FDCA production. This aligns with the observations of Eerhart et al. (2012)^24^, who found PEF (FDCA-based polymer) production that the FDCA purification stage is the most energy-intensive step in the entire production chain. Its energy consumption is 3–4 times that of the oxidation step, directly increasing fossil fuel consumption and carbon emissions. In terms of acidification (AC), the contributions from product separation and purification are also significant and comparable, at 41.24% and 51.55%, respectively, while product synthesis contributes only 7.22%. This aligns with the LCA findings of Lam et al. (2018)^58^, which identify the separation and purification stages—involving solvent evaporation and acidic wastewater discharge—as the primary sources of acidification potential in biomass-derived chemical production. In contrast, under the crystallization method (Fig. 8(b)), product separation emerges as the dominant contributor to all environmental impacts. It accounts for 81.54% of the human toxicity (HT) impact, with synthesis and purification/recovery contributing 7.69% and 10.77%, respectively. This highlights the need to prioritize optimization of the separation process to mitigate toxicity risks. Furthermore, the contributions of product separation and purification/recovery to abiotic depletion (AD) are closely aligned at 40.74% and 37.04%, indicating the necessity for integrated or synergistic strategies during process optimization. Notably, for fossil abiotic energy (FAE) and mineral abiotic energy (MAE) depletion, product synthesis contributes more than purification and recovery, accounting for 14.47% and 21.62%, respectively. This aligns with the findings of Eerhart et al. (2012)^24^, which indicate that the FDCA synthesis stage (HMF oxidation) consumes significant amounts of fossil-derived ethylene glycol (EG) when petroleum-based feedstocks are used. This further underscore the need for targeted efficiency optimization tailored to specific process steps based on the chosen production method.

**Fig. 9:**
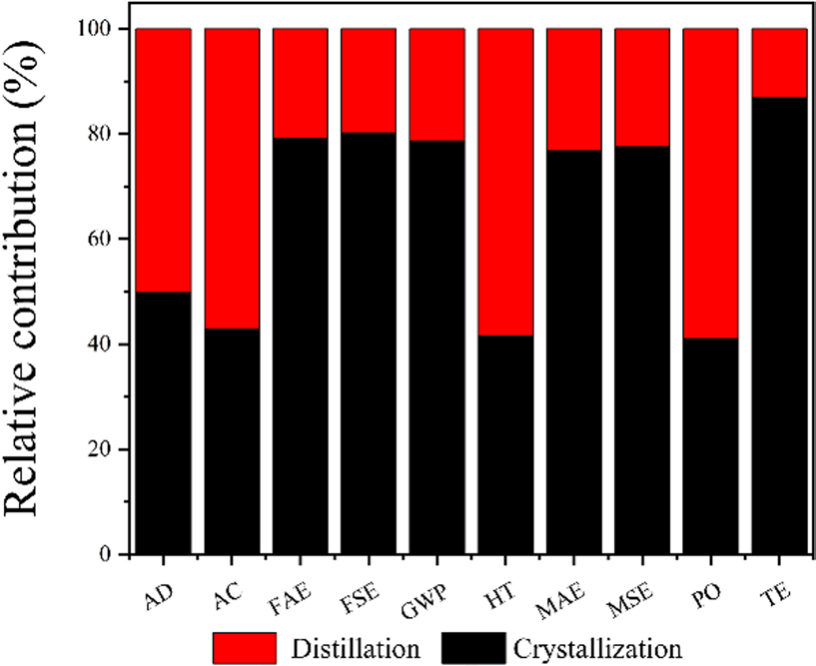
Relative contribution of distillation and crystallization for FDCA production.

A comparative analysis of the overall environmental impact of FDCA production via crystallization and distillation is presented in Fig. 9, revealing that the distillation method consistently results in greater environmental burdens across all assessed categories. Among these, terrestrial ecotoxicity (TE) exhibits the smallest difference, decreasing by only 12.99%, suggesting that TE is relatively insensitive to the choice of production method. The TE impact is primarily associated with the use of the Pt-ZrO_2_ catalyst, which, when introduced into the soil, may alter its physicochemical properties, inhibit microbial activity, and impair ecological functions. The presence of platinum (Pt) and zirconium (Zr) in soil can be absorbed by plants, negatively affecting their growth and potentially entering the food chain, where they may pose toxic risks to organisms at higher trophic levels. This aligns with the findings of Nuss et al. (2014)^60^in their LCA study of metals, which revealed that rare metals like platinum exhibit ecological toxicity 5 to 10 times greater than common metals such as aluminum. Therefore, developing and adopting green, low-toxicity, or biocompatible catalyst alternatives is crucial for reducing toxicity at the source. Terrestrial animals may encounter these heavy metals either through direct contact with contaminated environments or indirectly through consumption, leading to potential health issues. Therefore, the development and adoption of green, low-toxicity, or biocompatible catalyst alternatives are essential for reducing toxicity at the source. In addition to TE, the crystallization method achieves approximately 20% reductions in fossil abiotic energy (FAE), fossil secondary energy (FSE), global warming potential (GWP), mineral abiotic energy (MAE), and mineral secondary energy (MSE) compared to distillation, largely due to differences in the use of acetic acid. Acetic acid, when discharged into aquatic environments, can alter chemical characteristics and exert toxic effects on aquatic organisms, thereby disrupting ecosystem stability and biodiversity. Notably, in terms of global warming potential (GWP), the crystallization method yields 0.82 kg CO₂ eq/kg FDCA, while the distillation method results in 1.04 kg CO₂ eq/kg FDCA, representing a reduction of 21.29%. This finding aligns with the results reported by Eerhart et al. (2012)^24^. The GWP impact is mainly attributed to the electricity and thermal energy consumed during the process, which in this study are derived from fossil fuels; future substitution with renewable energy sources offers a viable path to mitigating this impact. Crystallization method yields 0.32 kg 1,4-DB eq/kg, while distillation method produces 0.75 kg 1,4-DB eq/kg, representing a 57.33% reduction in HT. This aligns with the findings of Lam et al. (2018)^58^that low-toxicity solvent crystallization processes reduce HT by 40%–60%. Crystallization yields 0.27 kg SO₂ eq/kg, while distillation produces 0.63 kg SO₂ eq/kg, representing a 57.17% reduction in AC. This aligns with Kim et al. (2022)^61^, who found that crystallization reduces AC by over 55% compared to distillation. The use of acetic acid and high-boiling-point solvents in distillation is the primary cause of elevated HT, consistent with Vioni et al.‘s (2024)^42^conclusion regarding the contribution of DMSO-water systems to high toxicity.

Additionally, the LCA in this study involves certain uncertainties and key assumptions: Regarding the system boundary, this study adopts a “factory gate-to-gate” boundary, excluding the production of raw materials (such as HMF) and the wastewater deep treatment stage. According to research by Goetz et al. (2023)^41^, incorporating the environmental impacts of HMF production would increase the GWP of both processes by 15% − 20%, yet the relative advantage of the crystallization method would remain unchanged. Based on hypothetical calculations by Goetz et al. for comparable wastewater scenarios, incorporating wastewater treatment in the future would increase the distillation route AC by approximately 25%, further widening the advantage of the crystallization method. Regarding parameter assumptions and sensitivity analysis, the power structure references China’s current average level (60% coal power + 40% renewable energy). If 100% renewable energy is adopted, based on HMF sensitivity analysis, the GWP output for both processes would decrease by over 60%, yet the crystallization method would still maintain a relative advantage.

In summary, the crystallization method significantly outperforms the distillation method across all key environmental impact categories: reducing GWP by 21.29%, HT by 57.42%, and FAE by 20.88%. Its environmental advantages align with findings from multiple studies, including those by Eerhart et al. (2012)^24^ and Kim et al. (2022)^61^. Only the TE difference is relatively small (12.99%). The core reason is that both processes employ Pt-ZrO₂ catalysts, whose metal components dominate the TE contribution due to their ecological toxicity (Nuss et al., 2014)^60^. Future efforts should focus on developing green catalysts such as sulfonated biochar (Lam et al., 2018)^58^.

### Future prospects

The industrialization of HMF and FDCA from biomass feedstocks in China remains in its early stages; however, notable progress has been achieved through supportive national policies and ongoing technological innovation^61–63^. Leading research institutions, including the Dalian Institute of Chemical Physics of the Chinese Academy of Sciences and the Qingdao Institute of Bioenergy and Process Research, have made significant advancements in the development of high-efficiency catalysts, the refinement of reaction conditions, and the enhancement of separation and purification technologies, efforts that collectively provide a strong foundation for future industrial-scale production. In parallel, several enterprises such as COFCO, Shandong Longli Bio, Henan Tianguan Group, and Jiangsu Jiutian High-Tech have begun establishing pilot projects aimed at scaling up HMF and FDCA production^64–67^. The implementation of national policy frameworks, such as the “14th Five-Year Plan” for bio-economic development, has further bolstered the sector by offering institutional support for the advancement of bio-based materials, thereby ensuring favorable conditions for accelerating industrialization efforts^65^.

In terms of policy support, the government’s strategic emphasis on promoting a green, low-carbon economy and advancing the development of bio-based materials has created a strong foundation for the high-value utilization of straw resources. From a technological perspective, continuous breakthroughs in key areas, including straw pretreatment, high-efficiency catalyst development, and the optimization of reaction processes, have further strengthened the groundwork for industrial-scale implementation. Additionally, China’s abundant straw resources ensure a stable and sufficient supply of raw materials for HMF production. Coupled with HMF’s significant potential as a platform compound in the fields of bio-based plastics, fuels, and pharmaceuticals, this has led to a rapidly growing market demand^65^. This demand is expanding at an average annual growth rate of 12%^65^.

However, the industrialization of HMF production still faces multidimensional technical and economic challenges that require critical examination: (1) Process scale-up bottlenecks: Current pilot-scale facilities (100–500 tons/year) exhibit specific energy (heating + electricity) consumption of approximately 35 kWh/kg HMF^61^. For industrial-scale plants producing tens of thousands of tons, energy consumption must be reduced below 20 kWh/kg to match the energy costs of fossil-based products^61^. Moreover, heat and mass transfer efficiency declines by 15%−20% during scale-up, resulting in an 8%−12% reduction in HMF conversion rates compared to pilot-scale levels^40^. Process stability requires further validation; (2) Energy Efficiency and Cost Pressures: The current production cost of straw-based HMF is approximately $11,806/ton. This is 41.7% higher than the market price of fossil-based alternatives (e.g., terephthalic acid) at approximately $8,333/ton^62^. Straw pretreatment accounts for 40% of costs (due to mechanical crushing and chemical reagent consumption), while catalyst depletion contributes 25%—cost structure optimization remains essential^62^; (3) Market competitiveness constraints: Bio-based FDCA’s market penetration remains below 5%^62^. This is primarily constrained by its price being 30%−40% higher than petroleum-based PET feedstock^63,64^. Furthermore, insufficient demand has emerged for scaled applications of downstream biodegradable materials (e.g., PEF), indicating inadequate supply chain synergy^63^. Looking ahead, these obstacles may be gradually overcome through continuous technological optimization and innovation, the coordinated development of the entire industrial chain, proactive efforts to secure policy and financial support, and the strengthening of international collaboration. With these combined efforts, the large-scale industrialization of HMF from straw in China is expected to advance steadily, ultimately becoming a vital driver in the transition toward a green economy and sustainable development^61,62^.

This study conducted a comparative analysis of the environmental impacts associated with the use of first-generation versus second-generation raw materials in the production of HMF, as well as an evaluation of two different processes, distillation and crystallization, for producing FDCA from oxidized HMF. Specifically, it assessed the resource consumption and environmental impacts of producing HMF and FDCA using crop straw as a raw material, providing a valuable theoretical reference for future large-scale industrial applications. Nevertheless, the study has certain limitations. It focuses solely on the environmental feasibility of producing HMF from corn straw, without incorporating an economic evaluation, which is essential for assessing commercial viability and should be prioritized in future research. Previous studies have indicated that a variety of waste biomass materials, such as bagasse, watermelon rind, apple pomace, and red algae, possess potential as feedstocks for HMF production, yet LCA data for these materials remain scarce^68,69^. Furthermore, this study considered only straw and fructose as feedstocks, underscoring the need to explore a broader range of biomass sources in subsequent investigations. Regarding FDCA production, only two thermochemical methods were examined, while alternative techniques such as electrochemical oxidation merit further LCA-based evaluation^70^. In summary, the task of developing high value-added chemicals from biomass is both challenging and ongoing, necessitating sustained collaboration among researchers, industry stakeholders, policymakers, and society at large. Such collective efforts are critical to overcoming technical barriers, optimizing industrial frameworks, and ultimately advancing the role of bio-based chemicals in achieving sustainable development and supporting the transition toward a green, circular economy.

## Conclusions

Straw, an agricultural waste, is environmentally preferred for HMF production. It outperforms fructose across all impact categories, reducing 87.73 kg CO₂ eq and 7.87 kg 1,4-DB eq emissions. It also mitigates ecotoxicity risks by 23.46%–27.83%. For FDCA synthesis, crystallization is far more sustainable than distillation. It cuts key environmental burdens by 20%–59.02% via lower energy consumption. Catalyst optimization is critical for reducing terrestrial ecotoxicity. Sensitivity analysis highlights actionable optimization pathways. 100% renewable energy reduces global warming by 74.56% for straw-based HMF production. Replacing DCM with green solvent GVL lowers human toxicity by 63.36%. These measures enhance environmental performance and technical feasibility, paving the way for industrial application. Overall, valorizing agricultural waste via straw-HMF and crystallization-FDCA routes aligns with sustainable development goals. This study provides pivotal insights for advancing biomass conversion technologies, supporting industrial scaling, and contributing to carbon neutrality. It highlights the transformative potential of green feedstocks and processes in the chemical industry.

## Data Availability

Data will be made available on request from the corresponding author [Q.-Y.L.].

## Abbreviations

LCA: Life Cycle Assessment
HMF: 5-Hydroxymethylfurfural
FDCA: 2,5-furandicarboxylic acid
DMSO: Dimethyl Sulfoxide
EPFG: Empty and Partially Filled Paddy Grains
DCM: Dichloromethane
DMA: N, N-dimethylacetamide
EtOAc: Ethyl Acetate
GHGs: Greenhouse Gases
GVL: γ-valerolactone
TOBr: Total Organic Bromine compounds
HFCS: High-fructose Corn Syrup
SPW: Sago Pith Waste
COFCO: China Oil & Foodstuffs Corporation
